# A Retrospective Study on Bile Culture and Antibiotic Susceptibility Patterns of Patients with Biliary Tract Infections

**DOI:** 10.1155/2022/9255444

**Published:** 2022-04-13

**Authors:** Chenwei Zhao, Shanshan Liu, Xue Bai, Jitao Song, Qiaowei Fan, Jing Chen

**Affiliations:** ^1^Department of Gastroenterology and Hepatology, The Second Affiliated Hospital of Harbin Medical University, Harbin 150000, China; ^2^Department of Gastroenterology and Hepatology, Shenzhen University General Hospital, Shenzhen 518000, China

## Abstract

**Aim:**

This study aimed to provide profiles of microorganisms isolated from bile and antibiotic susceptibility patterns of biliary tract infections (BTIs) in our center.

**Methods:**

A total of 277 patients diagnosed with BTIs at the Second Affiliated Hospital of Harbin Medical University from 2011 to 2018 were included in this study. Medical records were reviewed to obtain clinical and demographic data. Bile specimens were prepared through endoscopic retrograde cholangiopancreatography (ERCP), percutaneous transhepatic cholangiodrainage (PTCD), and percutaneous transhepatic gallbladder drainage (PTGD) under aseptic conditions. In those with positive bile culture results, blood cultures were concurrently conducted. The concordance of the results between bile culture and blood culture were also analysed.

**Results:**

Two hundred and sixty-seven bile cultures were positive, while 280 strains of micro-organisms were isolated. Among these, 76.8% were Gram-negative, 22.5% were Gram-positive and 0.7% were fungi. The most common microorganisms were *Escherichia coli*, *Klebsiella pneumoniae*, and *Enterococcus faecalis*. Gram-negative bacteria we tested were highly sensitive to ertapenem, imipenem, tigecycline, and amikacin. Gram-positive bacteria we tested were highly sensitive to tigecycline, teicoplanin, linezolid, vancomycin, and chloramphenicol. For the 44 patients with positive bile cultures, a blood culture was also performed. Among them, 29 cases yielded positive blood culture results. Among those cases with positive blood culture, 48.3% showed complete agreement with bile culture, 3.4% showed partial agreement, and 48.3% showed disagreement. The most common microorganisms in blood culture were the same as in bile culture. Additionally, the proportion of *Staphylococcus epidermidis* was significantly higher in blood culture (*P* < 0.05).

**Conclusion:**

Our study provided a comprehensive analysis of the bacteria distribution and drug resistance profiles in patients with BTIs in northern China. Further studies should be conducted to validate our findings.

## 1. Introduction

Biliary tract infection (BTI) is the bacterial infection in the biliary tract system, including situations such as acute or chronic cholecystitis and cholangitis [1, 2]. It is the common cause of intra-abdominal infections and life-threatening complications, especially in the elderly [3, 4], with high mortality rates of 9%–12% [5]. Appropriate antibiotics therapy played an important role in controlling infections and reducing mortalities, which indicates that rapid identification of the pathogens in BTIs and their antibiotic susceptibility profiles is essential in the initial management [4, 6]. As is known previously that blood culture is a convenient and effective way in detecting the causative organisms in BTIs, however, it remained negative in more than half of the cases [4]. With the development of biliary decompression techniques, such as endoscopic retrograde cholangiopancreatography (ERCP), percutaneous transhepatic cholangiodrainage (PTCD), and percutaneous transhepatic gallbladder drainage (PTGD), it seems that bile culture offered an opportunity for those with negative blood cultures. Several studies had explored the distribution of pathogens in bile cultures in the past years [7]. Based on these profiles, multiple guidelines on BTIs treatment had been published recommending antibiotic regimens that cover Gram-negative enterobacteriaceae, as well as an option of coverage of Gram-positive cocci and anaerobes [8].

However, in the past few years, there has been a worldwide increase of multidrug resistant (MDR) bacteria, such as extended-spectrum *β*-lactamase (ESBL)-producing Gram negatives and carbapenemase-producing (CPE) *Enterobacteriaceae* cultured from bile specimens [9, 10]. Besides, microorganisms showed both regional and temporal variations [11]; meanwhile, host factors such as pathogeny, immunodeficiency, or prior biliary interventions might also have impact on the selections of empirical antibiotics [7]. This might cause current empiric antibiotic therapy to be inadequate [8]. Therefore, the choice of antibiotic regimens remains a challenge to clinicians [12].

To the best of our knowledge, there is no agreement on the optimum empirical antibiotic regimens currently, and limited information is available regarding the antibiotic susceptibility profiles of the pathogens isolated from bile samples in northern China. Therefore, we designed this study to provide profiles of microorganisms isolated from bile and their antibiotic susceptibility patterns of BTIs in our center. We further compared the results from bile culture with results obtained through blood culture and discussed discrepancies from clinical perspective.

## 2. Materials and Methods

### 2.1. Patients and Study Design

We conducted this study at the Second Affiliated Hospital of Harbin Medical University. Patients diagnosed with BTIs at our center from 2011 to 2018 were included in this study. The following guidelines were referenced as diagnostic criteria of BTIs: (1) Guidelines for the Diagnosis and Treatment of Acute Biliary Tract Infections [13]; (2) Tokyo Guidelines 2018: Antibacterial Therapy for Acute Cholangitis and Cholecystitis [14]. The following patients were excluded: (1) lack of agreement; (2) those who did not meet the diagnostic criteria (including those with positive bile culture alone meanwhile present no clinical signs of infections, which we considered as asymptomatic biliary colonisation); (3) those who did not accept therapeutic ERCP or PTCD or PTGD; (4) patients with bacteraemia due to causes unrelated to BTIs.

A total of 277 patients were included in our study, bile specimens were prepared by intraoperative extraction through ERCP or PTCD or PTGD under aseptic conditions, medical records of these patients were reviewed, and the following information was obtained: age, sex, underlying diseases, comorbidities, clinical symptoms, primary disease, prior biliary interventions, and routes of bile collection. Besides, in those with positive bile cultures accepted blood cultures concurrently, we analysed the concordance of the results between bile culture and blood culture. The study was approved by the institutional review board at the Second Affiliated Hospital of Harbin Medical University on March 2nd 2015, with the approval number of 2015-研-065.

### 2.2. Bacteria Culture and Antibiotics Susceptibility

Specimen preparation in the present study were conducted in accordance with the National Guideline to Clinical Laboratory Procedures of China. Species identification and initial antibiotics susceptibility were identified by biochemical characterization using the VITEK2-Compact test (bioMérieux, Lyon, France). Antibiotic susceptibility was interpreted according to the Standardization of American Institution for Clinical Laboratory. Quality control strains, such as *Staphylococcus aureus* ATCC29213, *Enterococcus faecalis* ATCC29212, *Escherichia coli* ATCC25922, *Pseudomonas aeruginosa* ATCC27853, and *Klebsiella pneumoniae* ATCC700603, were obtained from the Clinical laboratory center of National Health and Family Planning Commission.

### 2.3. Statistical Analysis

Statistical analysis was performed through SAS 9.4 software. Categorical variables are presented as proportions or rates. Enumeration data were expressed as frequency. The comparison between groups was analysed by chi-square test or Fisher exact test. *P* < 0.05 was considered statistically significant.

## 3. Results

### 3.1. Clinical Characteristics of Patients with BTIs

The clinical characteristics of patients meeting the inclusion criteria are presented in Table 1. Patients with an age of ≥ 60 years were the majority of the participants (209/277; 75.45%), and cardiovascular disease was the most common underlying disease. Of the 277 patients, 185 (66.79%%) were male, while 92 (33.21%) were female. The majority primary diseases were benign (79.06%; *n* = 219), while 20.94% were malignant. In addition, 36 (13.00%) of these patients had undergone biliary intervention previously. Common manifestations presented by participants including abdominal pain (77.89%), jaundice (42.24%), and fever (41.52%). The bile specimens were collected via percutaneous procedures (PTCD or PTGD) in 184 (66.43%) and under endoscopic approach (ERCP) in 93 (33.57%) patients.

### 3.2. Distribution of Microorganisms Isolated from Bile Specimens

Distribution of microorganisms isolated from bile specimens is displayed in Table 2. Bile cultures were positive in 267 of 277 (96.4%), where nine had coinfections. A total of 280 strains of microorganisms were isolated.

Of all isolates, 215 were Gram-negative bacteria (76.8%), 63 were Gram-positive bacteria (22.5%), and two were fungi (0.7%). No anaerobes were detected in our study. The most common microorganisms were *Escherichia coli* (*n* = 73; 26%), *Klebsiella pneumoniae* (*n* = 44; 15.7%), *Enterococcus faecalis* (*n* = 34; 12.1%), ESBL-producing *Escherichia coli* (*n* = 12; 4.3%), *Pseudomonas aeruginosa* (*n* = 12; 4.3%), and *Enterobacter* (*n* = 12; 4.3%). *Escherichia coli* strains and *Klebsiella pneumoniae* strains had shown ESB004C-positive phenotype of 4.3% and 1.1%, respectively. Besides, two strains of yeast-like fungus were isolated (0.7%).

### 3.3. Antibiotic Susceptibility Patterns of Bacterial Isolates

**Figure 1:**
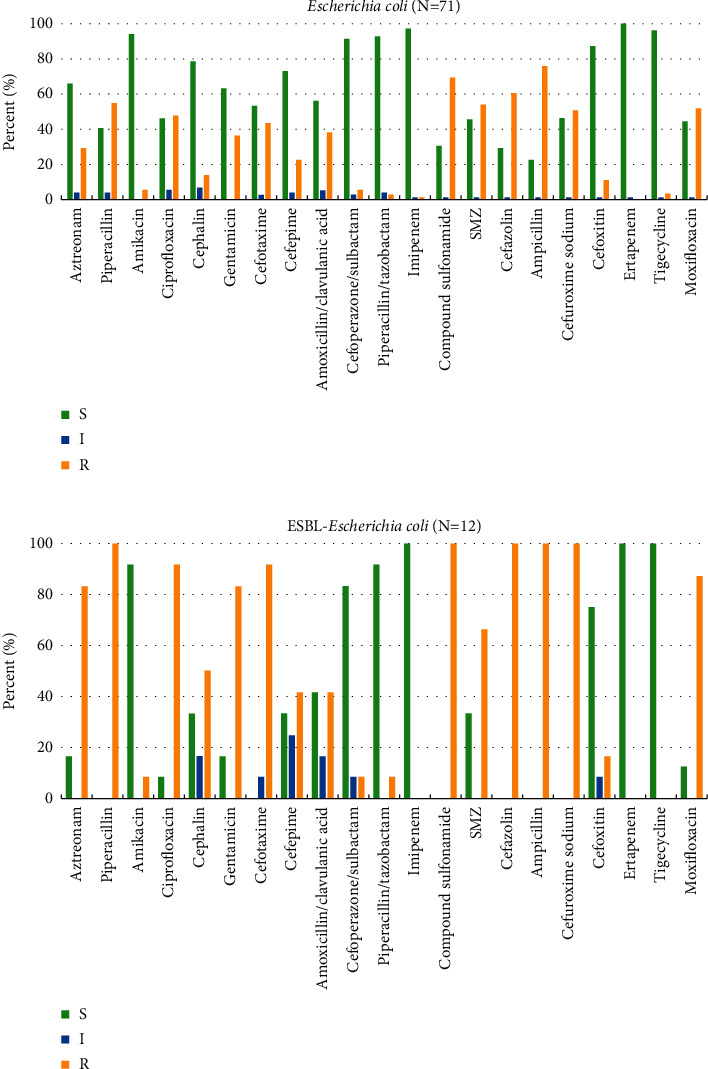
Antibiotic susceptibility patterns of *Escherichia coli* and ESBL-producing *Escherichia coli.* (a) *Escherichia coli.* (b) ESBL-producing *Escherichia coli.*

**Figure 2:**
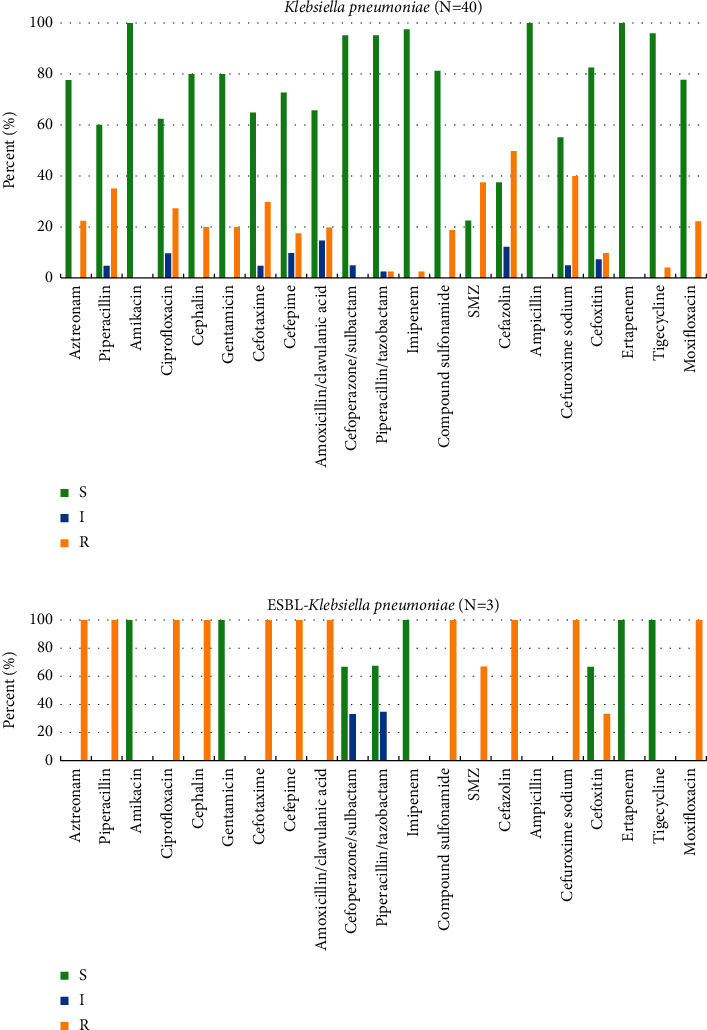
Antibiotic susceptibility patterns of *Klebsiella pneumoniae* and *ESBL-Klebsiella pneumoniae.* (a) *Klebsiella pneumoniae.* (b) ESBL-producing *Klebsiella pneumoniae.*

**Figure 3:**
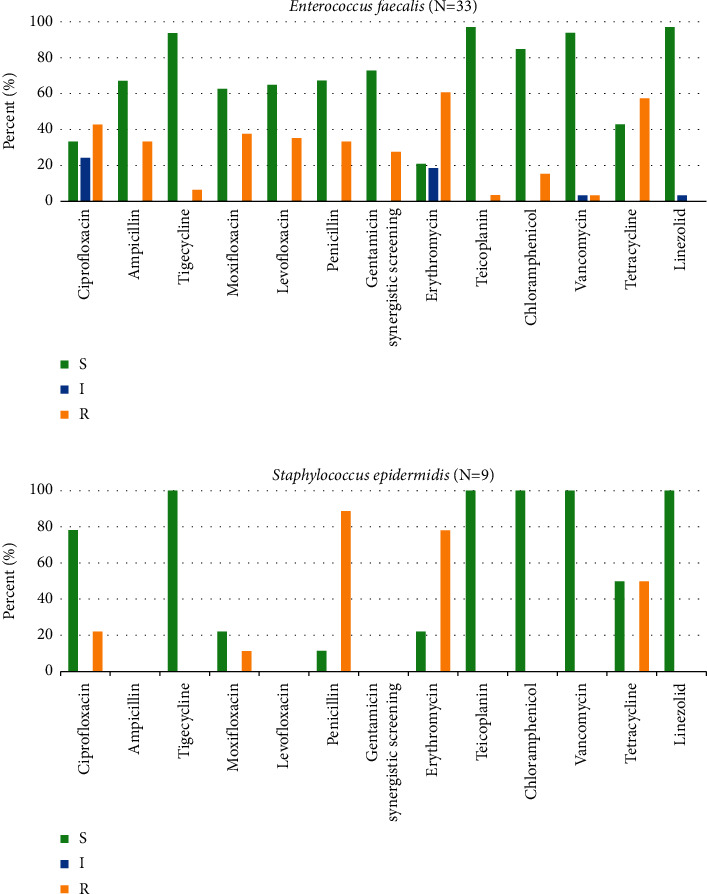
Antibiotic susceptibility patterns of *Enterococcus faecalis* and *Staphylococcus epidermidis*. (a) *Enterococcus faecalis*. (b) *Staphylococcus epidermidis*.

Antibiotic susceptibility testing was performed for most common isolated Gram-negative bacteria, *Escherichia coli*, *Klebsiella pneumoniae*, and their ESBL-producing phenotype, and the most common Gram-positive bacteria, *Enterococcus faecalis* and S*taphylococcus epidermidis*. The results are shown in Figures 1—3, respectively.

As can be seen from Figures 1(a) and 1(b), both *Escherichia coli* and ESBL-producing *Escherichia coli* were highly sensitive to ertapenem, imipenem, tigecycline, amikacin, and piperacillin/tazobactam, with high susceptibility rates above 90%. Piperacillin, compound sulfonamide, cefazolin, ampicillin, and cefuroxime sodium showed 100% efficacy against ESBL-producing *Escherichia coli* and only about 50% against *Escherichia col*i.

Antibiotic susceptibility patterns of *Klebsiella pneumoniae* are shown in Figure 2(a). *Klebsiella pneumoniae* was completely sensitive to amikacin, ampicillin, and ertapenem, and antibiotics with higher susceptibility rates include imipenem (97.5%), tigecycline (95.83%), cefoperazone/sulbactam (95%), and piperacillin/tazobactam (95%). As shown in Figure 2(b), ESBL-producing *Klebsiella pneumoniae* was also completely sensitive to amikacin, gentamicin, imipenem, ertapenem, and tigecycline. Besides, *Klebsiella pneumoniae* were more resistant to cefazolin, cefuroxime sodium, and SMZ with resistance under 50%. However, ESBL-producing *Klebsiella pneumoniae* was 100% resistant to more than half of the antibiotics we tested such as aztreonam, piperacillin, ciprofloxacin, and cephalin.

As seen in Figures 3(a) and 3(b), we only performed antibiotic susceptibility testing of two kinds of Gram-positive bacteria including *Enterococcus faecalis* and *Staphylococcus epidermidis*. Both *Enterococcus faecalis* and *Staphylococcus epidermidis* were highly sensitive to tigecycline, teicoplanin, linezolid, vancomycin, and chloramphenicol and were more resistant to erythromycin and tetracycline. Besides, ciprofloxacin also showed high activity against *Enterococcus faecalis*, while *Staphylococcus epidermidis* were resistant to penicillin.

In the present study, 44 patients with positive bile cultures accepted blood cultures at the same time. Of the 44 cases, 29 (65.9%) showed positive blood culture results.

Fourteen (48.3%) of these isolated the same organisms are like those found in the bile cultures. One (3.4%) showed partial agreement and 14 (48.3%) showed disagreement. The frequency of different microorganisms in positive bile and blood cultures was shown in Table 3. A total of 47 microorganisms were isolated from bile samples and 30 from blood samples. In general, the composition of the two results were similar, with 76.60% Gram-negative and 23.40% Gram-positive in the bile field, while 73.3% Gram-negative and 26.7% Gram-positive were present in the blood samples. Fungi was not isolated from bile or blood samples.

**Figure 4:**
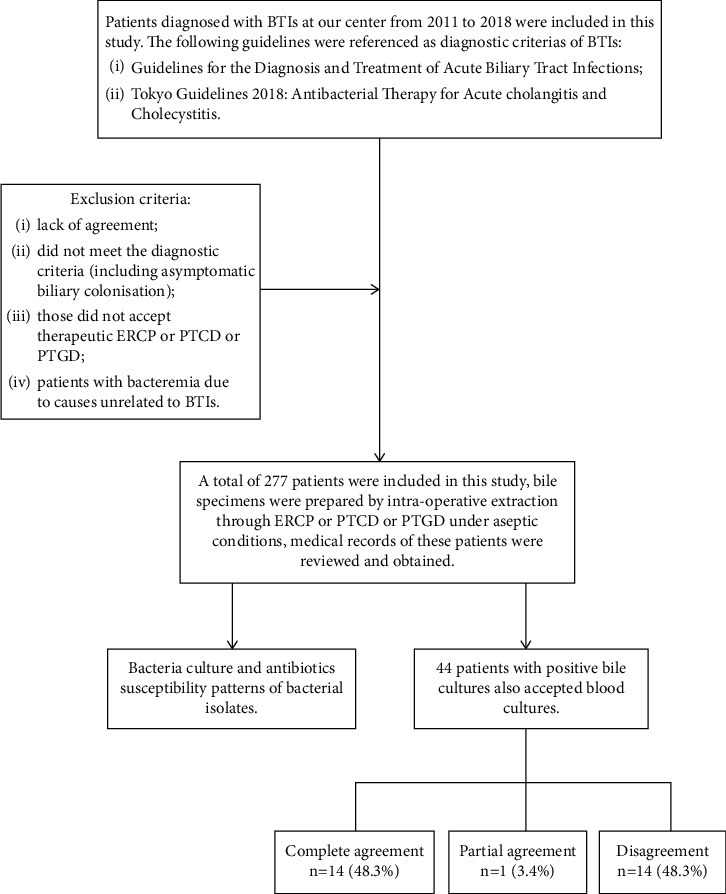
The flowchart and the comparison of organisms isolated from blood and bile.

The most common microorganisms were still *Escherichia coli* (*n* = 10; 33.4%), *Klebsiella pneumoniae* (*n* = 4; 13.4%), and *Enterococcus faecalis* (*n* = 4; 13.4%), same as bile cultures. However, the proportion of *Staphylococcus epidermidis* was significantly higher in blood culture (*P* < 0.05). The flowchart and the comparison of organisms isolated from blood and bile are briefly shown in Figure 4.

## 4. Discussion

BTIs usually reflect a severe life-threatening condition, which may result in sepsis and death [7]. It means the aggregation of bacteria in not only biliary tract but also blood and other organs, such as urinary tract [15–17]. Timely and appropriate application of antibiotics is crucial in the initial treatment. According to present clinical trials, the bioaccumulation of antibiotics was tissue-specific. The concentrations of antibiotics were diverse in different tissues [18–20]. Rational use of antibiotics should consider not only the antibacterial spectrum but also the distribution of antibiotics in different tissues. For example, it is better to choose antibiotics with higher levels in the cerebrospinal fluid for the treatment of bacterial meningitis, whereas antibiotics with higher levels in biliary tract should be selected to treat BTIs. Therefore, though bacteria could be the same in different infectious diseases, the antibiotic profile of bacteria should be different.

The importance of antibiotic regimens selection was highlighted by the fact that inappropriate empirical therapy is generally associated with increased mortality [7, 21, 22]. Inappropriate use of antibiotics, such as abuse of antibiotics, could also result in antimicrobial resistance, which threatens global health and imposes an enormous burden on society [23]. Under the pressure of antibiotics, bacteria developed resistance to multiple antibiotics through some mechanisms, including alteration of the active binding site of the antibiotic, restriction of antibiotic entry into the cell, active extrusion of the antibiotics by an efflux pump, and enzymatic inactivation of antibiotics [24]. According to the report of The Antimicrobial Use and Resistance in Australia (AURA) 2016, broad-spectrum antibiotics are more likely to contribute to antimicrobial resistance than narrow spectrum antibiotics [25]. Empirical antibiotic therapy of different clinicians usually represents an educated guess, based on the most possible specturm of causative pathogens and their expected antimicrobial susceptibility [26]. Although multiple guidelines had already provided a useful framework in general, standardized recommendations of antibiotic treatments are still widely lacking. Nevertheless, the selection of antibiotics is associated with various uncertainties such as the severity of BTIs [27], prior antibiotic exposure, and combined diseases. Besides, without the guidance of local microorganisms susceptibility records, it is difficult for attending physicians to make their choices. Therefore, we updated the local microorganism profiles of bile culture and antibiotic sensitivity patterns at our center, aiming to provide reliable evidences for clinical diagnosis and empirical antibiotic selection of BTIs in northern China.

Constantly with previous studies, most cases of BTIs were the elderly (75.45%) at our center [28]. Abdominal pain, fever, and jaundice were still the most frequently observed clinical manifestations in our study, with a presence of up to 77.98%, 41.52%, and 42.24%, respectively. In addition, our results showed a 96.4% positive rate of bile culture. Since the inclusion and exclusion criteria of our study were relatively strict, the rate of positive bile culture was higher than most studies reported before, with approximately 70% [28].

It has been reported that intestinal flora distribution such as *E. coli, Klebsiella pneumoniae,* and *Enterococcus faecalis* might be associated with BTIs [28–30]. Consistent with previous reports, the most common bacteria causing BTIs were still *E. coli, Klebsiella pneumoniae, and Enterococcus faecalis* in our study (Table 2). As to the frequency of different microorganisms in positive bile cultures, we found that Gram-negative bacteria (76.8%) were predominant, followed by Gram-positive bacteria (22.5%) and a small number of fungi (0.7%). However, one study from Germany showed that among all the bacterial isolates from bile culture, more were Gram-positive (57%), and *Enterococcus* species were predominant (494/1150 samples; 33%) [12], which was subsequently demonstrated by several studies [7, 12]. Recently, several studies had noticed that the frequency of Gram-negative enteric bacteria was declining slowly; meanwhile, Gram-positive *enterococci* was increasing slowly [28], and we did perceive an increasing trend of *Enterococcus* recently at our center, which was not displayed in this work. We considered that the cause of the phenomenon might be the conventional choice of empirical antibiotic therapies at our institution commonly covered Gram-negative bacteria, leading to the slight increase of Gram-positive *Enterococci*. The differences indicated that, in the future, more contemporary studies are required to characterize the current pathogen profile locally to optimize the therapeutic use of antibiotics in times [31, 32].

In general, the present study showed that both *Escherichia coli* and *Klebsiella pneumoniae* from bile culture were highly susceptible to ertapenem, tigecycline, imipenem, and amikacin with sensitivities exceeding 90%. A study conducted by Philippines and his colleges in 2011 showed that *Escherichia coli* and *Klebsiella pneumoniae* were highly susceptible to cefepime and aztreonam [33]. However, our results showed that both *Escherichia coli* and *Klebsiella pneumoniae were* resistant to cefepime and aztreonam, with resistance rates around 20%–30%. Besides, the resistance rates of the two bacteria were over 50% to the first-generation cephalosporins and around 20% to the fourth-generation cephalosporins, which were similar to the results of a study from South Korea [34]. The data in that study indicated that the drug-resistance rate was 14% to the fourth-generation cephalosporins and over 20% to the first-through third-generation cephalosporins [34].

As is known to all, the effect of penicillin derivatives and cephalosporins could be weakened due to extended-spectrum *β*-lactamases (ESBL). Recently, with the high incidence of ESBL-producing *Enterobacteriaceae*, the appropriate choice of empirical antibiotic therapy seems to be much more controversial. In view of this, physicians at our institution commonly hold the idea that penicillin is not a suitable choice of empiric antibiotic therapy for patients with BTIs, especially in cases of severe infections. Two types of ESBL-producing microorganisms isolated in the present study including *ESBL-Escherichia coli* and *ESBL-Klebsiella pneumoniae* were 100% sensitive to carbapenems and only 91.67% and 66.67% sensitive to piperacillin-tazobactam. It seems that carbapenems are the more appropriate choice with a broad empirical antibiotic coverage. However, a multicenter retrospective study in Singapore observed that the use of empiric carbapenems was associated with increased MDR and fungal infections compared with piperacillin-tazobactam [35]. Besides, it has been noticed that increasing usage of carbapenems might promote carbapenem-resistance, which is presently regarded as a major global public health problem. Therefore, it is widely advocated that carbapenems should be used in a more restrictive manner to minimize the selection pressure favouring growth of carbapenem-resistant bacteria [26]. Therefore, clinicians at our institution usually choose *β*-lactamase inhibitors including piperacillin/tazobactam as empirical antibiotics for mild BTIs and using third- or fourth-generation cephalosporins for moderate and severe biliary infections. Carbapenems were only used in situations when the previously mentioned treatment failed.

Confirmed by multiple studies, recently, we perceived an increasing trend of *Enterococcus* at our center [7, 12], indicating that Gram-positive *Enterococcus* might be another important pathogen of BTIs. This raised a question of whether the third-generation cephalosporines or ciprofloxacin commonly used in BTIs reflect an adequate choice for monotherapy [7, 12]. Consistent with previous studies [28], our results revealed an adequate susceptibility of both *Enterococcus faecalis* and *Staphylococcus epidermidis* to tigecycline, teicoplanin, linezolid, and vancomycin with susceptibility rates of more than 90%. Thus, for BTIs caused by *Enterococcus*, vancomycin is the drug of choice for empirical therapy at our center, while it was also on the recommended list of Tokyo Guidelines 2018 [14]. Besides, due to the presence of colonized bacteria in the bile duct, clinicians at our institution achieved a consensus that the purpose of antibiotic therapy is to prevent or relieve systematic inflammatory reactions rather than completely eradicating microorganisms in biliary tract.

In the present study, 44 patients with positive bile culture accepted blood culture at the same time, and the positive rate of blood culture was 65.9% (29/44). Gram-negative and Gram-positive bacteria accounted for 73.3% and 26.7%, respectively. The most common microorganisms in blood were still *Escherichia coli, Klebsiella pneumoniae*, and *Enterococcus faecalis.* The results are consistent with the findings of bile culture in the present study and are confirmed by several studies [4, 28, 36]. Blood culture seems to be a much more convenient way to detect pathogenic microorganisms without complicated procedures. However, previous studies revealed that more than half of the BTIs patients were negative for blood culture [4, 36], indicating that bacteria abundance in blood culture might be depressed. The results of our study also confirmed that the abundance of bacteria in bile culture is greater than blood culture. The fact that *Staphylococcus epidermidis* in blood culture is significantly higher than bile culture (*P*=0.027) also indicates that there might be a discrepancy between bile culture and blood culture. Besides, Rossolini and other researchers also proved that drug-resistant microorganisms such as *Pseudomonas* were much easier to detect from bile samples than blood [36, 37]. Therefore, blood culture alone as the reference of optimal empirical antibiotic therapy is inadequate and might result in growing mortality and healthcare costs for patients with BTIs.

Nowadays, the development of endoscopic and ultrasound-guided technologies such as ERCP of PTCD simplified the procedures obtaining bile samples, while bile samples can be collected directly from the sites of infections. Routinely collecting bile samples for culturing is of great help in pathogens detection and analysis of antibiotic resistance patterns and adjusting the current antibiotic therapy. Furthermore, the knowledge of local resistance rates may enable antibiotic stewardship strategies of clinicians [7]. Bile cultures now have retained their position as an early guide to antibiotic therapy for postoperative infections and are becoming the focus of revisited antibiotic stewardship strategies [9, 38]. Therefore, we recommend that bile samples should be routinely collected in patients with BTIs. Besides, former studies observed that microorganisms found in the positive blood cultures were usually found in bile samples concurrently [39]. However, the result of blood culture in our study showed almost half disagreement with bile culture, which is diverse from previous studies. The reasons for this discrepancy could be attributed to inadequate sample size, selection bias due to the numerous microorganisms found in bile, and the time interval between samples collections. Furthermore, some researchers found that the utility of bile culture might be dependent on the institutional antibiogram or regional biome for which it was created [9]. Therefore, more studies are still needed to further explore the value of bile cultures.

## 5. Conclusions

Delayed treatment of BTIs could lead to a series of serious consequences including septicopyemia, septic shock, multiple organ failure, or even mortality. Bacterial resistance increases continuously with the overuse of antibiotics, which make the treatment of BTIs more problematic. Therefore, the rational use and standard management of antibiotics are extremely necessary. Bacterial epidemiology of BTIs differs in different countries. According to previous reports from Germany, Gram-positive bacteria were predominant among all the bacteria isolates from bile culture. However, we found that Gram-negative bacteria were predominant in our center, which was consistent with some reports including surveillance report from China Antimicrobial Resistance Surveillance System [40, 41]. Based on the timely drainage and relief of biliary tract obstruction, *β*-lactamase inhibitors or carbapenems should be given for acute severe BTIs. Cefoperazone/sulbactam, piperacillin/tazobactam, imipenem, and ertapenem were recommended in such situations. Vancomycin or tigecycline should be used for patients' coinfection with Gram-positive bacteria. Moreover, antibiotics with high bile penetration rate should be used, such as cefoperazone/sulbactam and tigecycline, to ensure sufficient antibiotics concentration. In conclusion, our study provided a comprehensive analysis of the bacteria distribution and drug resistance profiles in patients with BTIs in northern China, which would help physicians to make better antibiotic selection. Further studies should be conducted to validate our findings to provide optimized treatment protocols for patients with BTIs.

## Figures and Tables

**Table 1:** Clinical characteristics of patients with BTIs.

Total study population	*N* = 277	
	%	(*n*)
Age (years)		
＜60	24.55	68
≥60	75.45	209
Sex		
Male	66.79	185
Female	33.21	92
Primary disease		
Benign	79.06	219
Malignant	20.94	58
Previous biliary intervention		
Yes	13.00	36
No	87	241
Underlying diseases		
Cardiovascular diseases	42.25	117
Diabetes	21.66	60
Manifestation		
Fever	41.52	115
Jaundice	42.24	117
Abdominal pain	77.98	216
Route of bile collection		
Endoscopic	33.57	93
Percutaneous	66.43	184

**Table 2:** Distribution of microorganisms isolated from bile specimens.

Microbial isolates		Total number	%

Gram-negative bacteria (*n* = 215;76.8%)	*Escherichia coli*	73	26.0
	*Klebsiella pneumoniae*	44	15.7
	*ESBL-Escherichia coli*	12	4.3
	Pseudomonas aeruginosa	12	4.3
	*Enterobacter*	12	4.3
	*Serratia odorifera*	8	2.8
	*Acinetobacter baumannii*	8	2.8
	*Klebsiella oxytoca*	7	2.4
	*Proteus mirabilis*	7	2.4
	*Citrobacter freundii*	6	2.1
	*Serratia marcescens*	4	1.4
	*Aeromonas hydrophila*	4	1.4
	ESBL-producing *Klebsiella pneumoniae*	3	1.1
	*Proteus vulgaris*	3	1.1
	*Morganella*	2	0.7
	ESBL-producing *Klebsiella oxytoca*	1	0.4
	*Proteus pannus*	1	0.4
	*Stenotrophomonas maltophilia*	1	0.4
	*Bacillus licheniformis*	1	0.4
	*Sphingomonas*	1	0.4
	*Aeromonas*	1	0.4
	*Moraxella catarrhalis*	1	0.4
	*C. faecalis*	1	0.4
	*Enterobacter aerogenes*	1	0.4
	*Bacillus subtilis*	1	0.4

Gram-positive bacteria (*n* = 63;22.5%)	*Enterococcus faecalis*	34	12.1
	*Staphylococcus epidermidis*	11	3.9
	*Streptococcus viridis*	6	2.1
	*MRSE*	3	1.1
	*Streptococcus bovis*	3	1.1
	*Corynebacterium diphtheriae*	3	1.1
	*Staphylococcus aureus*	2	0.7
	*Enterococcus faecium*	1	0.4
Fungus (*n* = 2;0.7%)	*Yeast-like fungus*	2	0.7

MRSE: methicillin-resistant *Staphylococcus epidermidis*.

**Table 3:** The frequency of different microorganisms in positive bile and blood cultures.

Pathogen		Bile culture *N* = 47		Blood culture *N* = 30		*χ* ^2^	*P*
		%	(*n*)	%	(*n*)		
*Gram-negative bacteria*	*Escherichia coli*	17.02	8	33.40	10	2.720	0.099
	*Klebsiella pneumoniae*	12.77	6	13.40	4	0.005	0.942
	*Enterobacter cloacae*	4.25	2	6.70	2	0.216	0.642
	*Pseudomonas aeruginosa*	4.25	2	3.30	1	0.042	0.838
	ESBL-producing *Escherichia coli*	6.38	3	3.30	1	0.346	0.557
	*Morganella*	0.00	0	3.30	1	1.587	0.208
	*Aeromonas hydrophila*	0.00	0	3.30	1	1.587	0.208
	*Proteus mirabilis*	8.51	4	3.30	1	0.808	0.369
	*Stenotrophomonas maltophilia*	0.00	0	3.30	1	1.587	0.208
	*Proteus*	2.13	1	0.00	0	0.647	0.421
	*Acinetobacter baumannii*	4.25	2	0.00	0	1.311	0.252
	*Corynebacterium diphtheriae*	2.13	1	0.00	0	0.647	0.421
	*Morganella*	2.13	1	0.00	0	0.647	0.421
	ESBL-producing *Klebsiella pneumoniae*	2.13	1	0.00	0	0.647	0.421
	*Sphingomonas*	2.13	1	0.00	0	0.647	0.421
	*Citrobacter freundii*	2.13	1	0.00	0	0.647	0.421
	Scented *Serratia*	2.13	1	0.00	0	0.647	0.421
	*Aeromonas*	2.13	1	0.00	0	0.647	0.421
	*Bacillus subtilis*	2.13	1	0.00	0	0.641	0.421
	Total	76.60	36	73.30	22	0.105	0.746

*Gram-positive bacteria*	*Enterococcus faecalis*	21.27	10	13.40	4	0.777	0.378
	*Staphylococcus epidermidis*	0.00	0	10.00	3	4.891	0.027
	Green grass *Streptococci*	0.00	0	3.30	1	1.587	0.208
	MRSE	2.13	1	0.00	0	0.641	0.421
	Total	23.40	11	26.70	8	0.105	0.746

MRSE: methicillin-resistant *Staphylococcus epidermidis*.

## Data Availability

The data that support the findings of this study are available from the corresponding author upon reasonable request.
